# Supplementary material for: Concise gene signature for point‐of‐care classification of tuberculosis
Source: EMBO Mol Med. 2015 Dec 18;8(2):86–95. doi: 10.15252/emmm.201505790 (PMC4734838; doi:10.15252/emmm.201505790)
Supplement: Supplementary file 1 — Appendix [file EMMM-8-086-s001.pdf]

Appendix

Table of Content

Appendix Figure S1 – PCA analysis on Indian dataset page 2

Appendix Figure S2 – Performance of signatures on external validation sets page 3

Appendix Figure S3 – Performance on independent microarray datasets page 4

Appendix Table S1 – Independent cohorts for RT-PCR validation page 5

Appendix Table S2 – Ranked gene list of 15-gene RF model page 6

Appendix Figure S1.

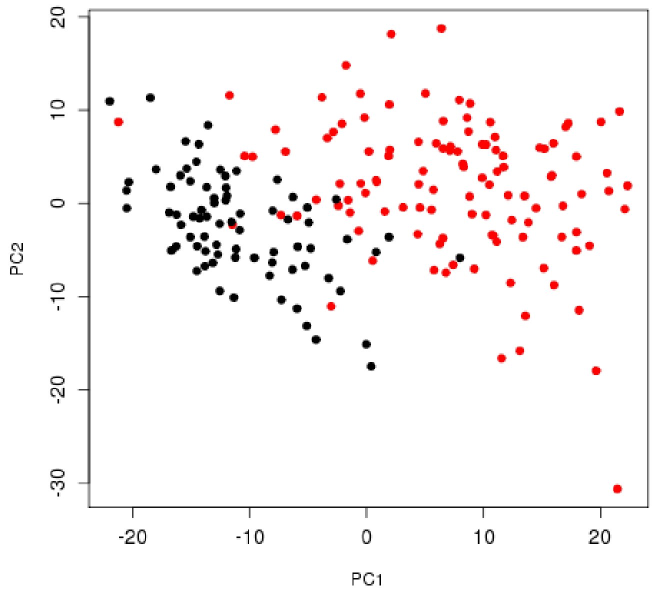

Principle component analysis on the total dataset. The 189 samples roughly segregate into the pre-defined TB (red) and control groups (black).

Appendix Figure S2.

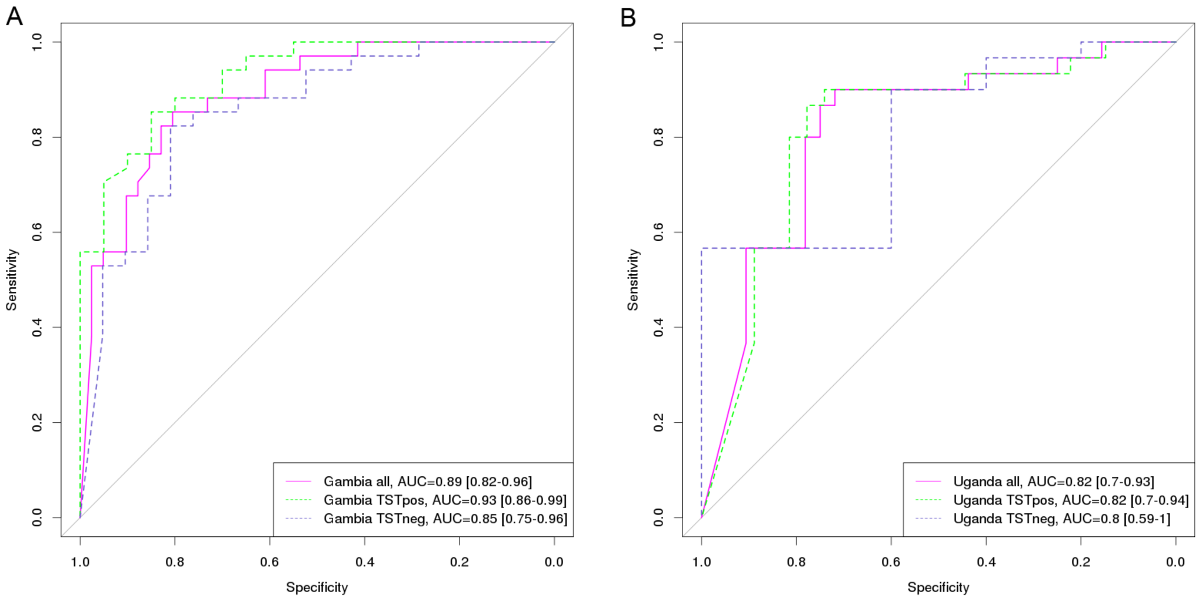

Performance of the 4-gene model in two validation cohorts (The Gambia and Uganda) based on RT-PCR –derived gene expression data. Solid lines represent classification power between TB and all controls, dashed lines the classification between TB and the latently infected (TSTpos) or uninfected (TSTneg) controls.

Appendix Figure S3

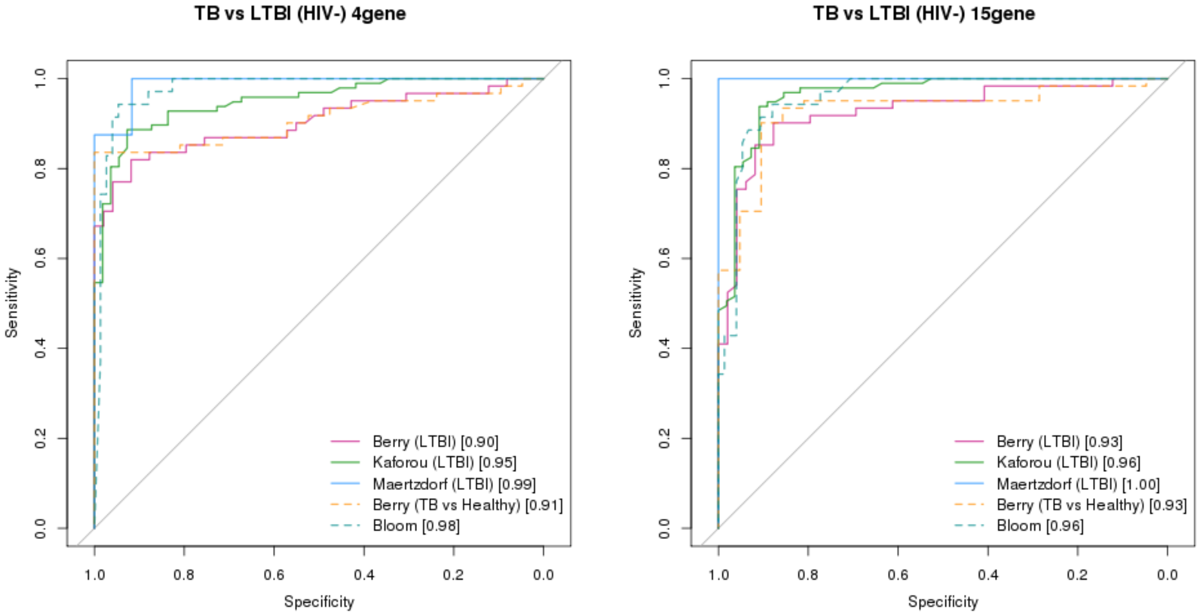

Classification performance of the 4-gene and 15-gene set models on external microarray datasets in HIV- cohorts. Solid lines represent classification power between TB and latently infected (LTBI) controls, dashed lines the classification between TB and uninfected individuals.

Appendix Table S1.

	SET	TB	LTBI	CTRL
**India**	Training and test sets	113	56	20
**The Gambia**	Blind validation set	34	20	21
**Uganda**	Blind validation set	30	24	8

Number of TB patients, latently infected (LTBI) and uninfected (CTRL) healthy controls in the RT-PCR validation cohorts.

Appendix Table S2.

Ranke	GeneSymbol	Description
1	CNIH4	Cornichon Family AMPA Receptor Auxiliary Protein 4
2	GBP1	Guanylate-binding protein 1
3	P2RY14	Purinergic receptor P2Y, G-protein coupled, 14
4	PCNXL2	Pecanex-like 2 (Drosophila)
5	CD274	CD274 molecule; programmed death-ligand 1 transmembrane protein
6	FCGR1C	Fc fragment of IgG, high affinity Ic, receptor (CD64), pseudogene
7	GBP5	Guanylate-binding protein 5
8	S100A8	S100 calcium-binding protein A8
9	ID3	Inhibitor of DNA binding 3, T lymphocyte-associated
10	DHRS9	Dehydrogenase/reductase (SDR family) member 9
11	FBXL5	F-box and leucine-rich repeat protein 5
12	FAM26F	Family with sequence similarity 26, member F
13	BATF2	Basic leucine zipper transcription factor, ATF-like 2
14	CD96	CD96 molecule; T cell activation-induced membrane protein
15	FCGR1A	Fc fragment of IgG, high affinity Ia, receptor (CD64)

Gene panel of the 15-gene random forest model built on the 100 sample training set (Indian cohort).

Genes are listed in decreasing order of importance.
