# Supplementary material for: Concise gene signature for point‐of‐care classification of tuberculosis
Source: EMBO Mol Med. 2015 Dec 18;8(2):86–95. doi: 10.15252/emmm.201505790 (PMC4734838; doi:10.15252/emmm.201505790)
Supplement: Supplementary file 2 — Expanded View Figures PDF [file EMMM-8-086-s002.pdf]

# Expanded View Figures

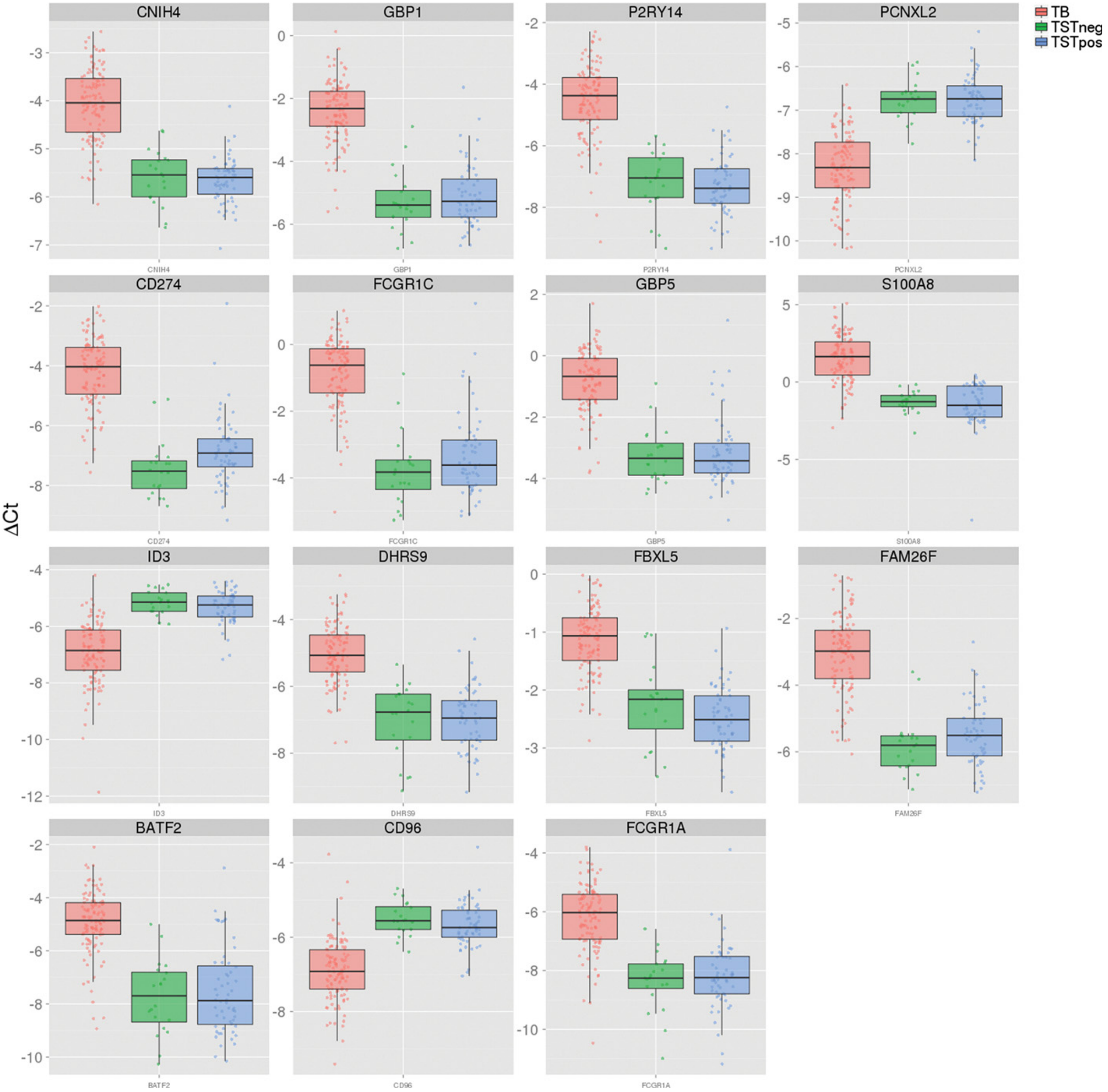


**Figure EV1. Relative expression levels.**
Box plots showing relative expression of genes from 15-gene model in Indian RT–PCR dataset. Displayed are inverse Δ*Ct* values (zero − Δ*Ct*), such that higher values indicate higher expression levels for each sample.

© 2015 The Authors

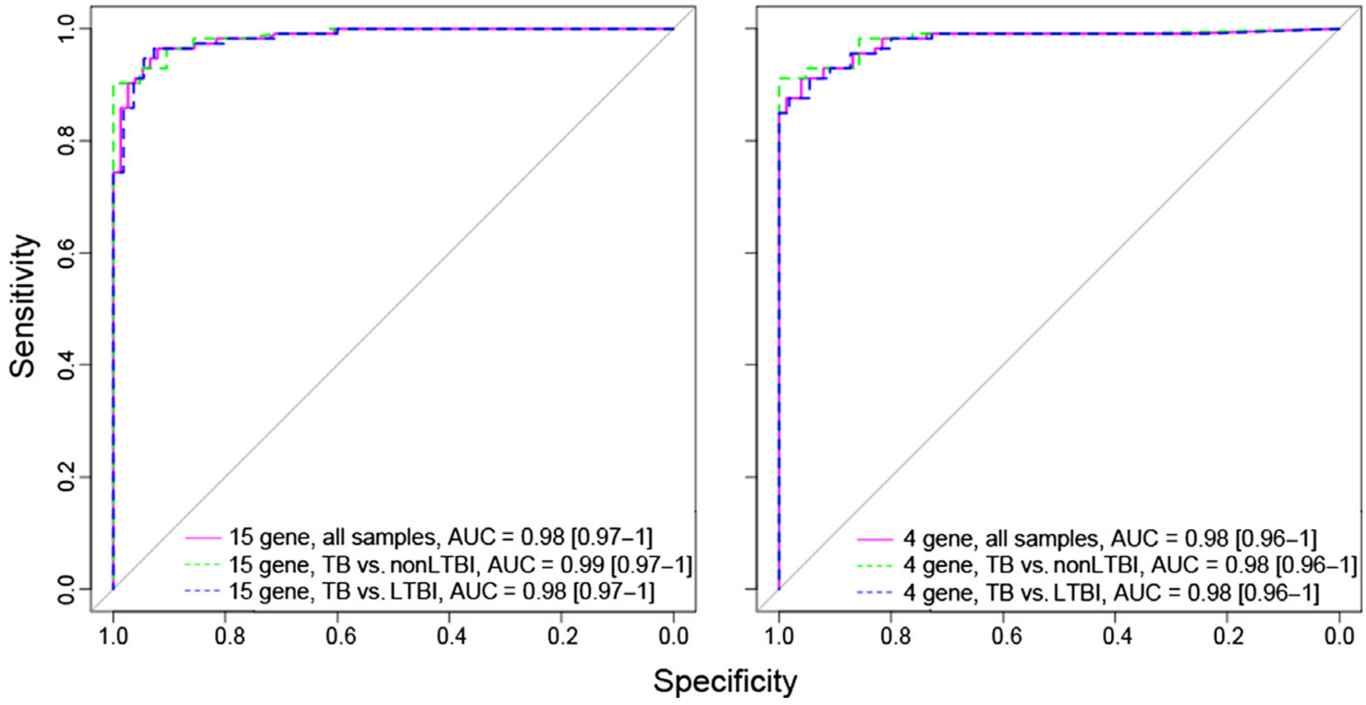


**Figure EV2. Classification performance of models.**
ROC curves showing the performance of the 15- and 4-gene models built on the combined training and test datasets. Shown is classification power between tuberculosis (TB) and all controls, and between TB and the LTBI or uninfected (non-LTBI) controls separately.

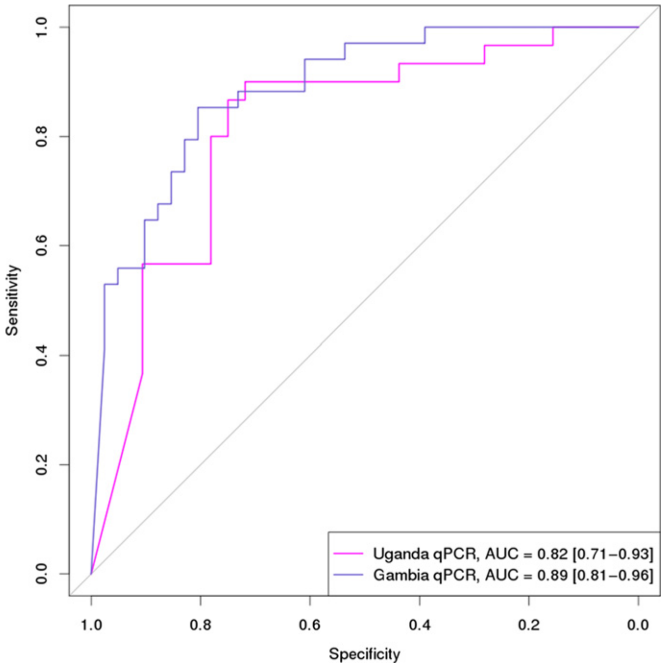


**Figure EV3. 4-gene model performance on external RT–PCR datasets.**
Performance of the 4-gene model in two validation cohorts (The Gambia and Uganda) based on RT–PCR-derived gene expression levels.

 © 2015 The Authors

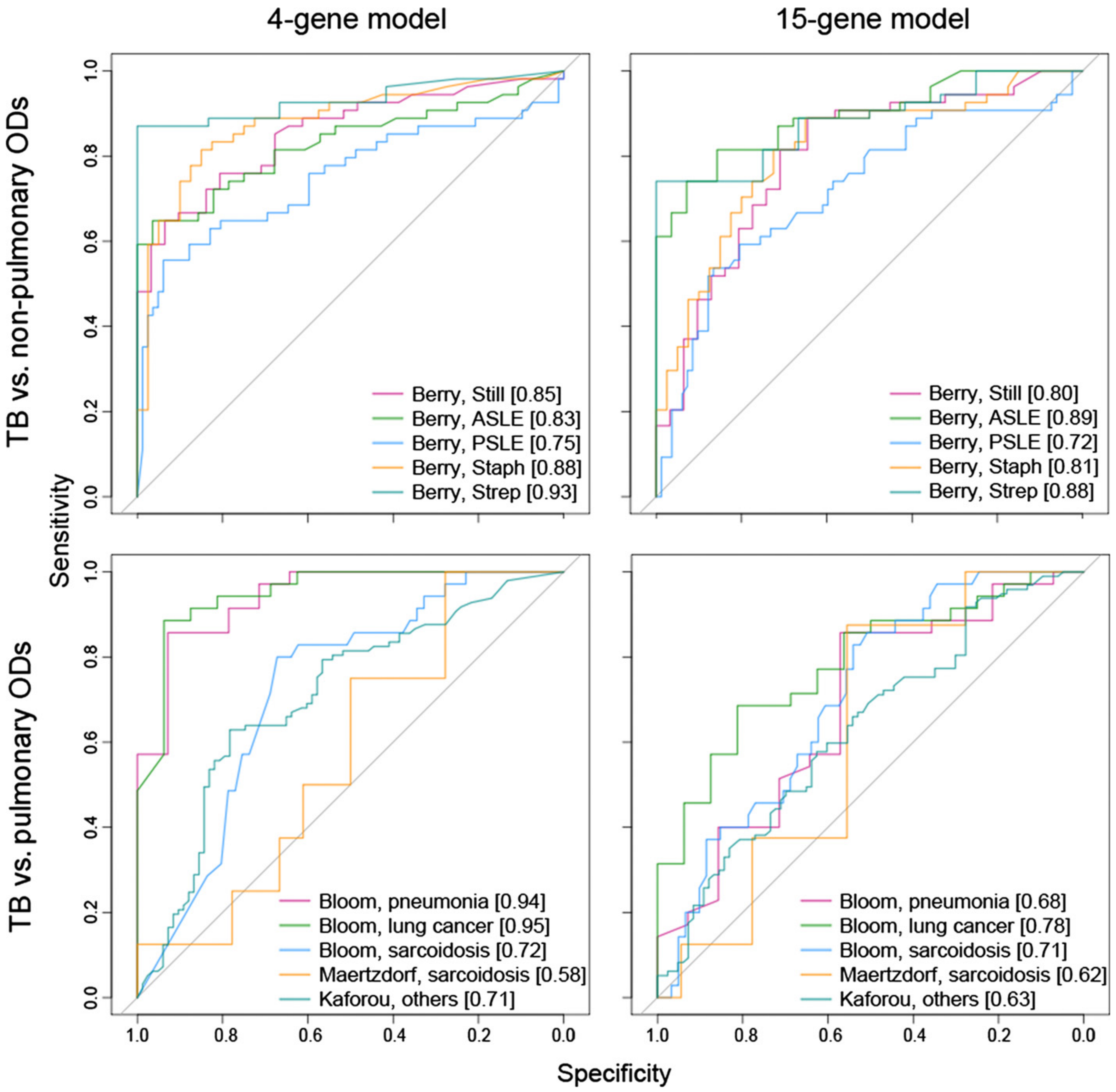


**Figure EV4. Classification performance on tuberculosis (TB) and other diseases (ODs).**
Performance of our 4- and 15-gene signatures classifying TB from other diseases in external validation datasets (ODs). Disease abbreviations in Berry dataset (Berry *et al*, 2010): Still, Still's disease; ASLE, adult systemic lupus erythematosus; PSLE, pediatric SLE; Staph, *Staphylococcus aureus*; Strep, Group A *Streptococcus*.

© 2015 The Authors
